# In vivo acute oral toxicity assessment of novel histone deacetylase 2 inhibitor

**DOI:** 10.1186/s40360-025-01040-9

**Published:** 2025-11-28

**Authors:** Padmini Pai, Rachel Savio D’Mello, Shruthi Nayak, Pallavi Rao, Srinivas Oruganti, Kapaettu Satyamoorthy, Babitha Kampa Sundara

**Affiliations:** 1 Department of Biophysics, Manipal School of Life Sciences, Manipal Academy of Higher Education, Manipal, 576104 Karnataka India; 2 Manipal School of Life Sciences, Manipal Academy of Higher Education, Manipal, 576104 Karnataka India; 3 Dr. Reddy’s Institute of Life Sciences, University of Hyderabad Campus, Gachibowli, Hyderabad, 500046 India; 4 Shri Dharmasthala Manjunatheshwara (SDM) University, Manjushree Nagar, Sattur, Dharwad, 580009 India

**Keywords:** Acute oral toxicity, BALB/c mice, Histone deacetylase inhibitor, Hydroxamic acid, Lethal dose, OECD 423

## Abstract

Histone deacetylases (HDACs) are enzymes responsible for removing acetyl groups from histone proteins, resulting in chromatin condensation and the repression of genes. They regulate the expression of genes, the cell cycle, and multiple cellular processes. Hydroxamic acid is a well-recognized moiety characterized by its potent zinc-binding ability, making it an effective inhibitor of HDACs. A novel hydroxamic acid-based molecule, N^1^-(2,2’-bipyridin-6-yl)-N^8^-hydroxyoctanediamide (compound 3B), was previously synthesized, and the anticancer properties of the compound were examined in vitro in our laboratory. No prior toxicological study has been performed on this compound. Therefore, the current investigation focused on the acute oral toxicity of compound 3B in female BALB/c mice, adhering to OECD 423 guidelines. In this study, compound 3B was given orally at 300 mg/kg body weight (b.w.) or 2000 mg/kg b.w. The food consumption and body weight of the mice did not differ significantly between the control and treated groups. Variations were observed in the levels of a few of the biochemical markers. Histopathological examination revealed inflammatory infiltration and lesions in a few vital organs. The comprehensive investigation revealed that compound 3B exhibited moderate toxic effects at a relatively high dosage of 2000 mg/kg in few organs and caused alterations in biochemical markers; however, it did not result in any mortality, indicating that the LD_50_ value exceeded 2000 mg/kg. Compound 3B can be administered at concentrations less than 2000 mg/kg for subsequent studies.

**Graphical Abstract:**

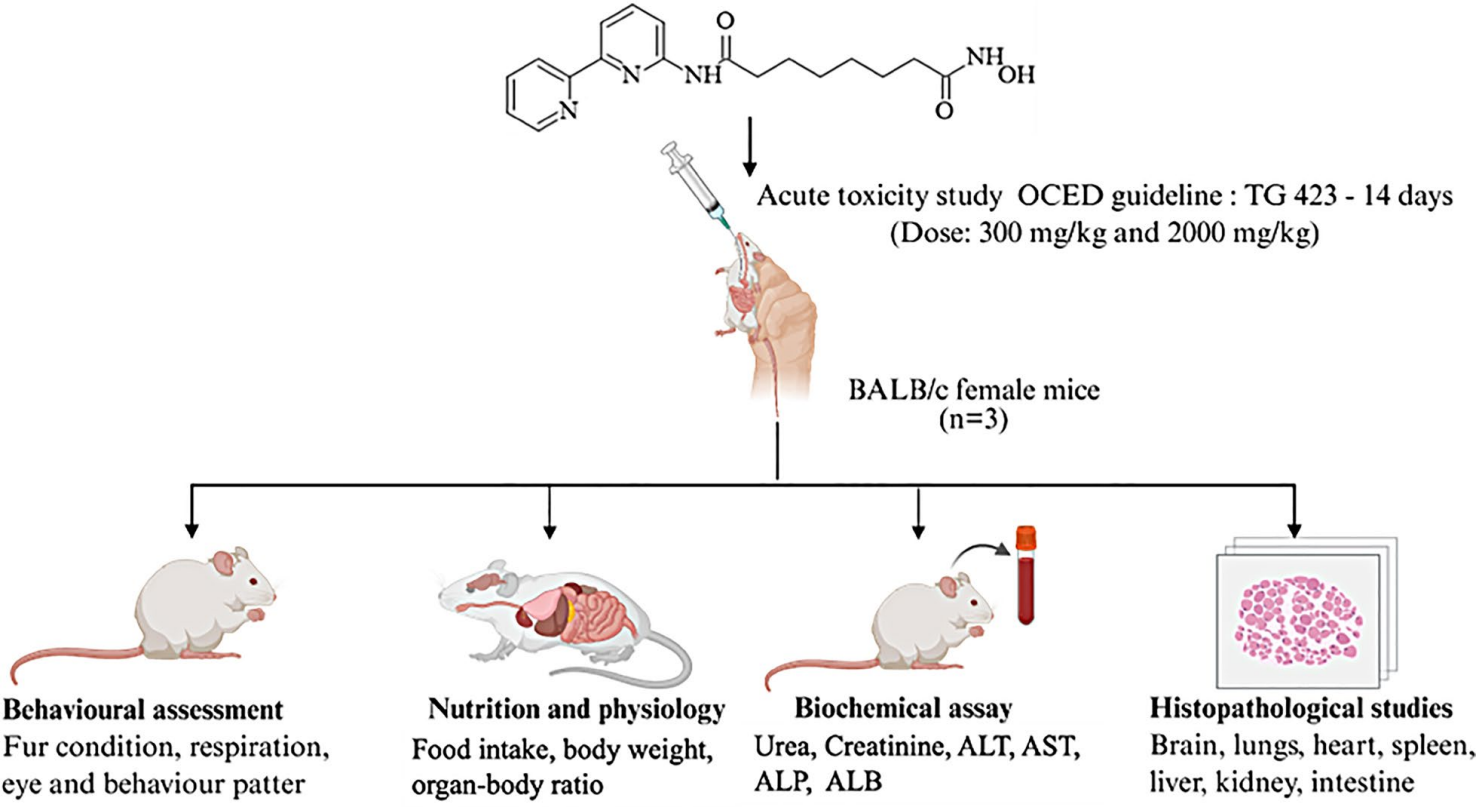

## Key highlights

The toxicity profile of compound 3B was studied in female BALB/c mice as per the OECD 423 guidelines.

Compound 3B was given through oral gavage at dosages of 300 mg/kg and 2000 mg/kg

Biochemical marker levels and histopathological examination revealed moderate toxic effects of compound 3B at 2000 mg/kg

Compound 3B is classified under category 5 (lowest toxicity) according to the OECD 423 guidelines.

## Introduction

Cancer is a global health problem that affects millions of people worldwide. Approximately 10 million deaths were reported worldwide in 2020 [1]. Surgery, radiation therapy, and chemotherapy are some of the approaches used to treat cancer [1]. In recent decades, numerous anticancer agents have been developed, yet each has its own challenges and does not offer a completely effective solution [2]. Drugs targeting epigenetic writers and erasers for cancer therapy first started with the concept of inhibiting individual targets with drugs such as HDAC inhibitors (HDACis), histone methyltransferase inhibitors, and DNA methyltransferase inhibitors. The primary obstacle is combatting the issue of drug resistance [3, 4]. Hence, there is a constant need for the development of more precise, specific, effective and safer drugs. Histone deacetylases (HDACs) are frequently overexpressed in various cancers, contributing to the transcriptional repression of tumor suppressor genes. Consequently, HDACis are commonly employed in the treatment of hematological malignancies and a few solid tumors [5]. Hydroxamic acid, known for its strong Zn^2^^+^chelating ability, exhibits high binding affinity and potent inhibitory effects, making it a widely adopted zinc-binding moiety in drug design [6, 7].

For a medication to enter clinical practice, preclinical toxicity evaluations are crucial. These investigations employed verified methods and suggested animal models. Correlating animal reactions to those in humans is the ultimate objective of toxicity research [8]. The hazardous characteristics of the substance can be evaluated, ranked, and categorized on the basis of the globally harmonized system (GHS) for classifying substances that show acute toxicity on the basis of information provided in the Organization for Economic Cooperation and Development (OECD) guidelines420, 423, and 425. According to OECD Guideline 423 [9], the method categorizes the test chemical into classes determined by defined LD_50_ cut-off values [10].

**Fig. 1:**
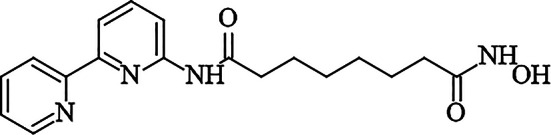
Structure of N^1^-(2,2’-bipyridin-6-yl)-N^8^-hydroxyoctanediamide (compound 3B)

Previously, in our laboratory, we identified a novel HDAC2 inhibitor, N^1^-(2,2’-bipyridin-6-yl)-N^8^-hydroxyoctanediamide (compound 3B) (Fig. 1). To date, pharmacological data on compound 3B have been derived from in vitro studies, with no toxicological evaluation conducted in rodent models. This study aimed to assess the acute oral toxicity of compound 3B in BALB/c mice in accordance with the OECD 423 guidelines. The results are anticipated to yield essential understanding of the safety profile of compound 3B when administered orally, thereby aiding its possible advancement as a new HDAC inhibitor for cancer therapy.

## Materials and methods

### Chemical structure

A novel hydroxamic acid derivative, compound 3B, was synthesized and characterized in our laboratory. This compound has been patented by the Manipal Academy of Higher Education, Manipal (Patent ID No. 202441019540), and preliminary in vitro studies support the potency of compound 3B. The structure of compound 3B is given in Fig. 1.

### Animals

Female BALB/c mice with an average weight of 28.55 ± 3.38 g were bred and maintained in the institutional animal facility (Central Animal House Facility, Manipal Academy of Higher Education, Manipal) under controlled standard laboratory conditions. Ethical approval for animal use in this study was obtained from the Institutional Animal Ethics Committee (IAEC/KMC/46/2025), Kasturba Medical College, Manipal, India. The animals were kept in a controlled environment of 23 ± 2 °C, a humidity of 60 ± 5%, and light/dark cycles of 10 hours and 14 hours, respectively. The mice had free access to food and water. Animal care and handling were carried out in accordance with OECD 423 guidelines.

### Experimental design

**Fig. 2:**
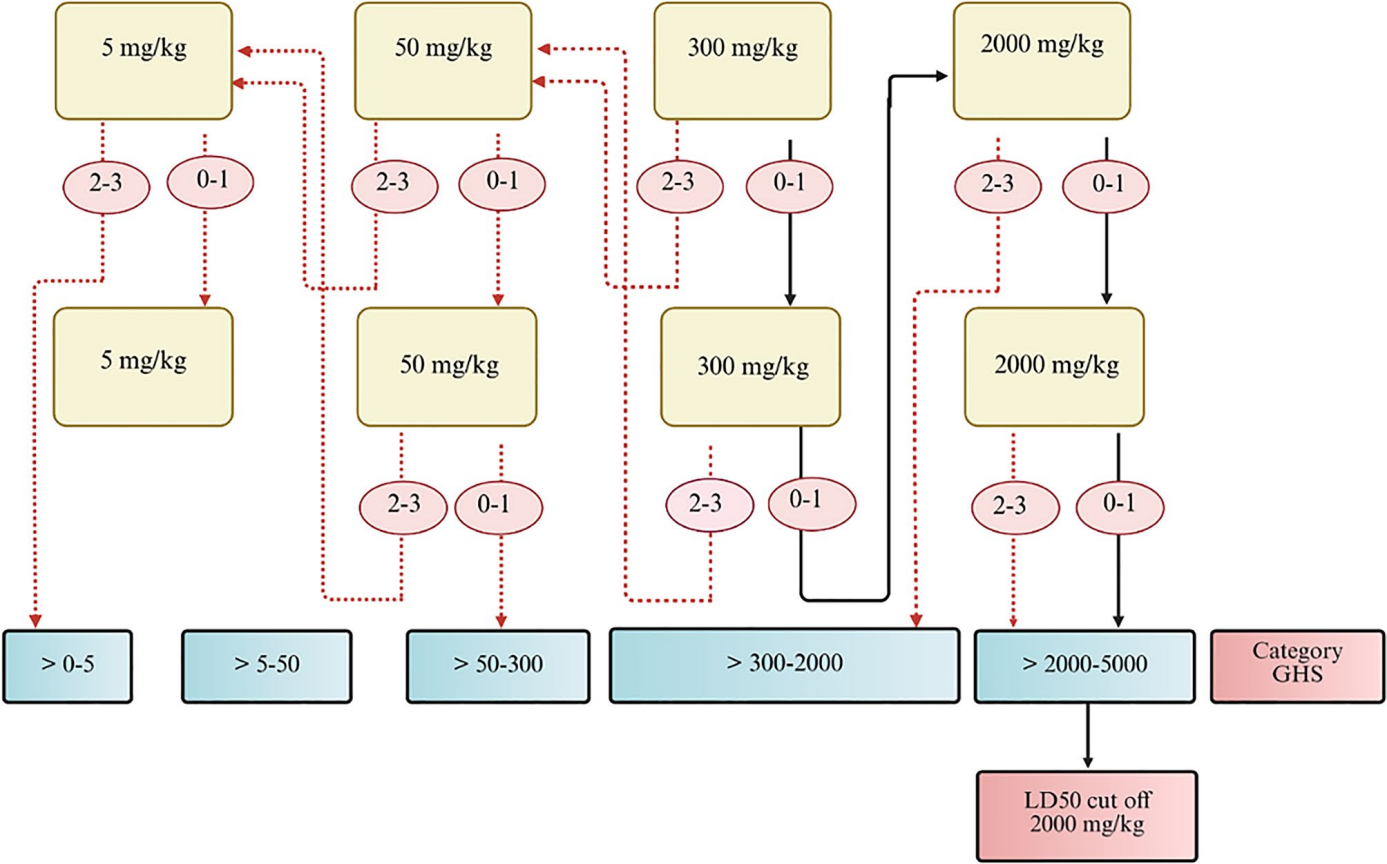
Schematic representation of the experimental design for determining the LD_50_ cut-off value for compound 3B [9]

Toxicological evaluation of compound 3B was conducted in vivo following the OECD 423 (OECD-423), [9] guidelines for acute oral toxicity. Following these guidelines, three female BALB/c mice were utilized for each dose step. The acute toxicity range of compound 3B was determined on the basis of the mortality status of the mice, which aids in classifying the test compound. The OECD guidelines suggest starting with a 300 mg/kg b.w. dosage when no prior toxicological data for the compound exist to comply with ethical considerations. Fig. 2 presents the detailed experimental design for determining the LD₅₀ cut-off value of compound 3B in accordance with OECD guideline 423 [9]. The corresponding table summarizes the experimental groups used in the study. The mice were fasted for 1–2 hours before dosing. The compound 3B-treated group received oral administration of compound 3B via gavage at doses of 300 mg/kg and 2000 mg/kg. The suspension consisted of 10% DMSO (Merck, Sigma‒Aldrich) and 10% Cremophor (Merck, Sigma‒Aldrich), and it was dissolved with the help of a probe sonicator (Sonics, Vibrio cell). The control group was administered a combination of 10% DMSO and 10% Cremophor, excluding the test substance. A volume of 1 ml/100 g b.w. was administered, and the volume was altered on the basis of the weight of the mice. Food was given 1 hour after drug treatment. The mice were closely observed for the first 24 hours and subsequently for the next two weeks. The mice were weighed, and food consumption was monitored daily. The fur condition, behavior, respiration, ocular and mucous membranes, urine, and excretion were observed daily. Biochemical parameters, organ-to-body weight ratios, and histological assessments were performed on day 14 of the study. Three mice were taken per step, with a 3-day interval between each step, to observe the toxicity before administration of the next dose. The animals were anaesthetized with ketamine–xylazine administered intraperitoneally (ketamine 80 mg/kg; xylazine 10 mg/kg). Upon confirmation of anaesthesia, the animals were euthanized via transcardial perfusion with phosphate-buffered saline followed by 4% paraformaldehyde [11–13]. Histopathological analysis of different organs and biochemical assessment of various parameters were carried out after euthanization (Table 1).

**Table 1:** Experimental treatment groups for OECD 423 acute toxicity studies of compound 3B

Group No	Dosage	No. of mice
Group 1	10% DMSO and 10% Cremophor	3+3
Group 2	Compound 3B, 300 mg/kg	3+3
Group 3	Compound 3B, 2000 mg/kg	3+3

### Biochemical analysis

The animals were anaesthetized with ketamine–xylazine administered intraperitoneally (ketamine 80 mg/kg; xylazine 10 mg/kg). Upon confirmation of anaesthesia, the animals were euthanized via transcardial perfusion with phosphate-buffered saline followed by 4% paraformaldehyde [11–13]. On day 14 of the experiment, after the mice were anaesthetized, blood samples were taken. A capillary was used to collect blood from the retro-orbital region. Clot activator (BD vacutainers) vacutainers were used for the collection of blood samples. After the blood had clotted, the tubes were spun at 2000 × g for 10 mins, and the serum was extracted. The alanine aminotransferase (ALT) content was estimated via the UV kinetic method with an ERBA SGPT kit. Albumin (ALB) was determined via the BCG dye method via an albumin kit. Alkaline phosphatase (ALP) activity was analysed via the pNPP DEA kinetic method via an alkaline phosphatase kit. Creatinine (CREA) was measured via Jaffe’s kinetic method via a creatinine kit. Aspartate aminotransferase (AST) was assessed via the UV kinetic method via the ERBA SGOT kit, and urea (UR) was estimated via the urease method via the ERBA Urea (BUN) kit. All the analysis was carried out using Erba EM 200.

### Histopathological study

Vital organs (brain, heart, lungs, stomach, kidney, spleen, liver and intestine) were extracted from the mice that had been sacrificed and stored in a 4% formalin solution. The organs were dehydrated with different concentrations of isopropanol, immersed in xylene, and embedded in paraffin wax. Four-micron sections were cut with a microtome (Leica RM2125 RTS) and stained with hematoxylin and eosin (H&E) to observe the pathological changes in the organs via an LX-500 LED trinocular research microscope (Labomed), and images were taken with a MiaCam CMOS AR 6pro microscope.

### Statistical analysis

Body weight, dietary intake, the organ-to-body weight ratio, and biochemical parameters were assessed via GraphPad Prism 8.0 software. The observed results were statistically analysed and are presented as the means ± SDs. One-way and two-way ANOVA were used to assess the significance between the datasets, followed by Dunnett’s multiple comparison test. A *p* value of ≤ 0.05 was considered statistically significant.

## Results

### Observation of behavior pattern

After compound 3B was administered at doses of 300 mg/kg and 2000 mg/kg, all the mice were individually monitored for 14 days. No notable alterations in skin condition, respiration, ocular or mucous membranes, behavior, urine or excretion were detected. Throughout the experiment, no abnormalities or deaths were noted in the mice.

### Body weight and food intake

**Fig. 3:**
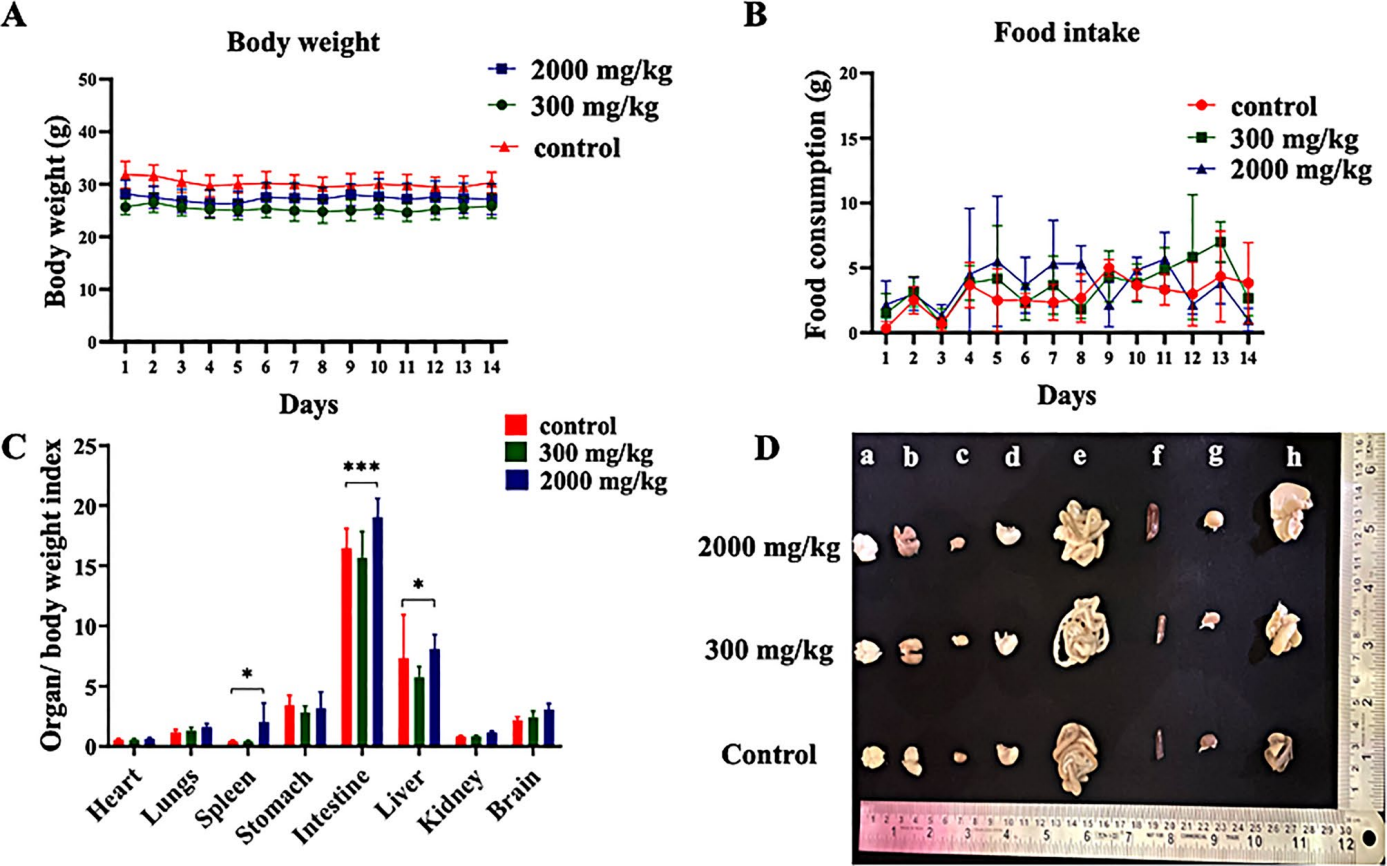
Effects of the administration of compound 3B on (**A**) body weight, (**B**) food intake, (**C**) the organ/body weight index and (**D**) images of different organs from the control, treatment (300 mg/kg), and treatment (2000 mg/kg) groups; (**a**) body weight, (**b**) lungs, (**c**) heart, (**d**) stomach, (**e**) intestine, (**f**) spleen, (**g**) kidney, and (**h**) liver. All the data are reported as the means ± SDs; *n* = 6 per group. Two-way anova was performed, followed by Dunett’s test. The significance levels observed are * *p* < 0.05 and ***p < 0.001. Organ-to-body weight index = (organ weight/body weight × 100)

All the treatment groups maintained relatively stable body weights throughout the 14-day toxicity study, as shown in Fig. 3A. Furthermore, daily food consumption slightly varied; however, as shown in Fig. 3B, no statistically significant differences were noted between the control and treatment groups.

### Organ to body weight index

Organs such as the brain, heart, lungs, intestine, stomach, spleen, kidneys, and liver were weighed. For every mouse, the organ-to-body weight index was determined. As shown in Fig. 3C, the ratios of the heart, lungs, brain, kidneys, and stomach among the different treatment groups did not significantly differ. The groups of mice that received compound 3B at a dosage of 2000 mg/kg, however, exhibited significant differences in the organ-to-body weight indices of the intestine, spleen, and liver. Fig. 3D presents representative images of organs from three groups: the control group and the two test groups.

### Biochemical analysis

Biochemical assays have been performed to assess toxicity in the liver and kidneys. Table 2 shows that there was no significant difference in the CREA or UR across all the experimental groups. ALT and ALB levels were not significantly different among the three different groups in terms of liver function. AST levels increased at 2000 mg/kg, but the difference was not statistically significant. ALP levels significantly decreased at higher concentrations.

**Table 2:** Effects of the administration of compound 3B on renal and liver function tests

Organs	Control	300 mg/kg	2000 mg/kg
UR (mg/dl)	42.00 ± 24.33	22.00 ± 2.65	36.33 ± 1.53
CREA (mg/dl)	0.27 ± 0.29	0.63 ± 0.12	0.23 ± 0.06
ALT (U/L)	68.00 ± 33.65	40.00 ± 36.72	32.67 ± 6.43
AST (U/L)	69.67 ± 18.61	41.00 ± 32.23	105.00 ± 6.93
ALP (IU/L)	209.00 ± 60.36	146.33 ± 20.11**	157.00 ± 8.66 *
ALB (g/dl)	2.10 ± 0.10	2.17 ± 0.06	2.27 ± 0.12

UR: urea; CREA: creatinine; ALT: alanine aminotransferase; AST: aspartate amino transferase; ALP: alkaline phosphatase; ALB: albumin. All data are reported as the mean ± SD for *n* = 3 per group. The differences between the control and treated groups were analyzed by Two-way ANOVA, followed by Dunnett’s test. The significance levels observed are *p < 0.05, **p < 0.01, and in comparison to control group values

### Histopathological analysis

**Fig. 4:**
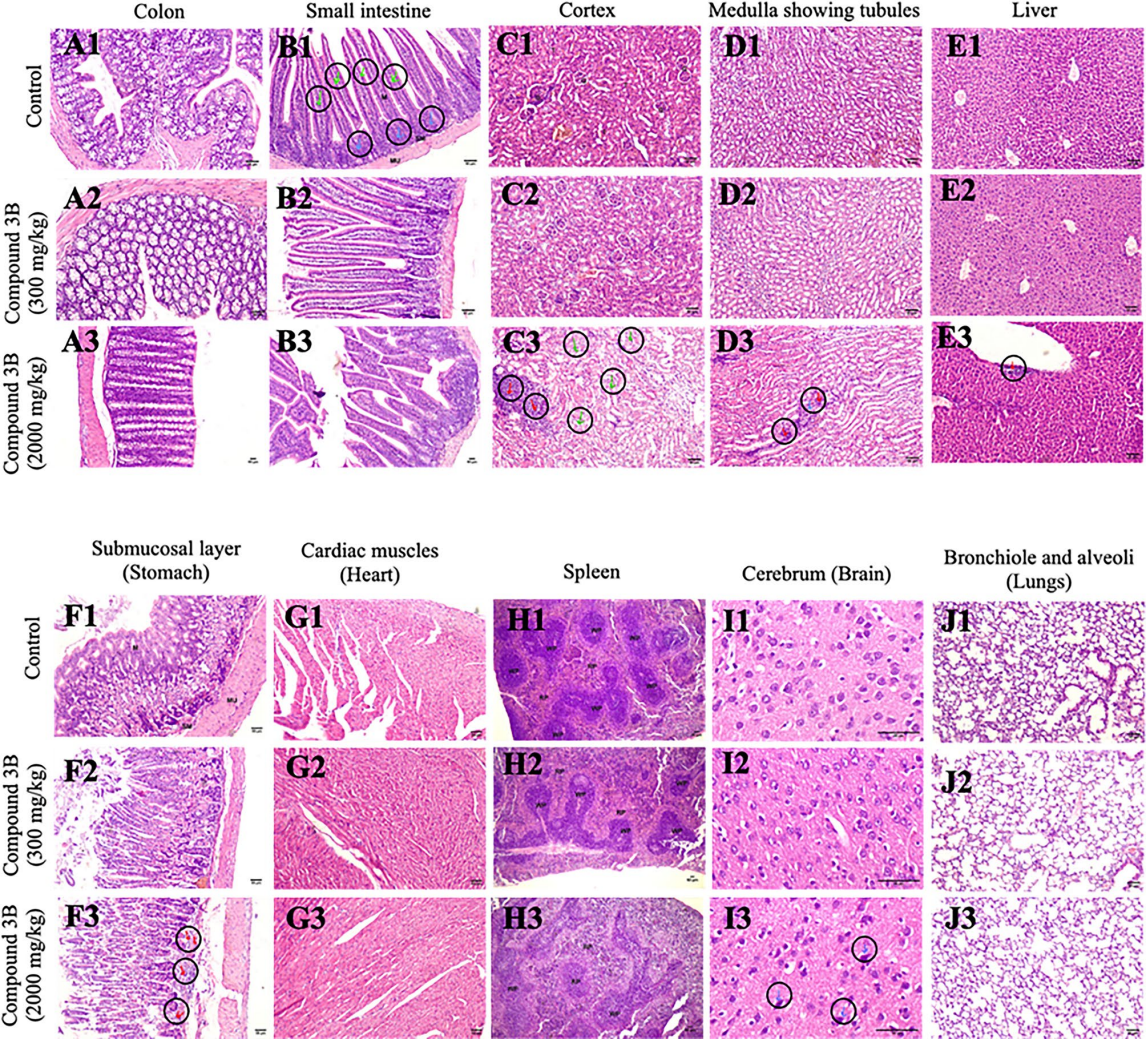
H&E stained tumor tissue sections from control and treated mice, showing histological evaluation of major organs including control, treatment (300 mg/kg) and treatment (2000 mg/kg); (**a**) colon, (**b**) small intestine, (**c**) cortex, (**d**) medulla (kidney), (**e**) liver (100x), (**f**) stomach, (**g**) heart, (**h**) spleen, (**i**) cerebrum, and (**j**) lungs. Mucosa - m, submucosa - sm, white pulp - wp, red pulp - rp, tubules - t, glomerulus - g, inflammatory infiltrate - red arrow, eroded mucosa- blue arrow, degenerated tubules - green arrow, necrosed tubules- blue arrow; data represented as *n* = 3, mice were treated with compound 3B at two different doses: 300 mg/kg and 2000 mg/kg. Scale: 50 µm; spleen-40x, cerebrum-400x, all other organs-100x

Histopathological examination of major organs revealed that the morphology and color of the liver, lungs, kidneys, heart, and spleen were generally maintained across all test groups. In the colon (Fig. 4A), histological analysis revealed an intact architecture without any discernible abnormalities. Similarly, the small intestine (Fig. 4B) maintained normal morphology. However, notable inflammatory infiltrates were observed in the group receiving a 2000 mg/kg dose. Renal histology (Fig. 4C, D) revealed well-preserved glomeruli, Bowman’s capsules, and renal corpuscles across most groups, whereas the 2000 mg/kg group displayed signs of tubular degeneration, necrosis, and inflammatory infiltration. The hepatic sections (Fig. 4E) clearly revealed portal triads and central veins without evidence of necrosis or hemorrhage. The gastric mucosa (Fig. 4F) appeared structurally intact, although inflammatory infiltration was again noted in the 2000 mg/kg group. The myocardial architecture of the cardiac tissue (Fig. 4G) retained organized myocardial fibres across all the groups. Splenic histology (Fig. 4H) revealed a reduction in the cellularity of white pulp in both test groups, alongside observations of extramedullary hematopoiesis and acute inflammatory cells. Cerebral sections maintained normal morphology overall, with the exception of a few degenerated neurons observed in the 2000 mg/kg group. Pulmonary histological analysis (Fig. 4I) confirmed preserved alveolar and bronchiolar structures, although the 300 mg/kg group presented hemorrhage, whereas the 2000 mg/kg group presented inflammatory infiltration. Fig. 4JCompared with that in the control group, acute inflammatory infiltration was detected in the test group.

## Discussion

The acute toxic class method, which is an approach aimed at classifying substances according to their acute oral toxicity, was introduced in OECD guideline 423. Using small groups of animals (usually three per step), this method employs known dose levels of 5, 50, 300, and 2000 mg/kg body weight to ascertain the toxicity class of a substance. On the basis of the mortality observed at each dose, additional testing may involve dosing more animals at either the same or different dose levels [10]. In the present study, Compound 3B, a novel hydroxamic acid derivative, was evaluated for its acute oral toxicity at doses of 300 mg/kg and 2000 mg/kg. In our previous study, we investigated the effects of compound 3B on neuroblastoma. In our previous study, we investigated the effects of compound 3B on neuroblastoma. The compound demonstrated anticancer activity with an IC₅₀ value of 4.44 µM. Compound 3B inhibited HDAC1 and HDAC2 at 0.44 μM and 1.94 μM, respectively. Western blot analysis further revealed increased levels of acetylated H3K9, confirming HDAC inhibition [14] and patenting (Patent ID No. 202441019540). In the present study, with respect to all doses, no mortality was observed at either dosage, indicating that the LD50 was greater than 2000 mg/kg. A similar study by Liu and colleagues revealed that almond hull powders did not significantly affect the organ-to-body weight index of mice across all treatment groups, which encompasses vital organs, such as the liver, kidney, heart, lungs, and spleen [15]. Similarly, in our study, compound 3B did not affect behavior, body weight or dietary consumption at either dosage (300 mg/kg or 2000 mg/kg). From a statistical perspective, the 300 mg/kg dosage did not significantly affect the ratios of the heart, lungs, brain, kidneys, and stomach. However, for the 2000 mg/kg group of mice, there were significant differences in the intestine, spleen, and liver organ-to-body weight indices. Another study by Bedi and colleagues revealed that evaluating liver and kidney function through markers such as ALT, AST, ALP, ALB, CREA and UR can help determine the toxicity of a compound [16]. Our investigation revealed that kidney and liver function tests did not reveal any statistically significant differences among the different treatment groups. AST and ALT levels increase significantly, which may be attributed to the destruction of liver cells within a toxic environment [17]. Another study by Yang and colleagues reported that, owing to their high concentrations in liver cells, ALT and AST are often used as more precise indicators for measuring suspected liver cell damage. Elevated ALT and AST levels can indicate liver damage [18]. Our research demonstrated that ALT and AST levels did not differ significantly among the treatment groups. Lala and colleagues demonstrated that elevated ALP levels often indicate biliary tract obstruction linked to cholesterol liver disease [19]. The slight variations in ALP levels were considered incidental and unrelated to the test substance. This finding indicates that our compound, 3B, does not affect metabolic activity or renal functions.

It was proposed that histopathological studies offer supportive evidence for biochemical and hematological observations [20]. Histopathological analysis of major organs revealed dose-dependent alterations, with inflammatory infiltrates, tubular degeneration, necrosis, and hemorrhage being prominent in the high-dose groups (2000 mg/kg). These findings indicate the potential for dose-dependent toxicological effects, highlighting the importance of dosage optimization for safe therapeutic applications. This is the first study to investigate OECD toxicity guidelines for evaluating the LD_50_, mortality, and other key toxicological parameters of compound 3B. Acute in vivo toxicity studies provide preliminary safety data; however, these studies are associated with limitations. Studies have been conducted in a single animal species, and because of interspecies metabolic differences, the findings may not reliably predict human responses [21, 22]. The observation period was short (up to 14 days), thereby leading to the neglect of long-term chronic toxic effects. In addition, only a single dose level is tested, which restricts the ability to establish a full dose–response relationship or LD₅₀ with precision [23]. Further research is needed to comprehensively assess the long-term effects of this compound.

### Conclusion

In accordance with OECD guideline 423, a 14-day acute oral toxicity study was conducted. The study revealed that compound 3B falls under Category 5, with an estimated LD_50_ above 2000 mg/kg body weight. Although slight signs of toxicity were observed at the 2000 mg/kg dose, mortality was not observed. Thus, the LD_50_ of compound 3B is considered to be greater than 2000 mg/kg.

## Data Availability

No datasets were generated or analysed during the current study.

## Abbreviations

ALB: Albumin
ALP: Alkaline phosphatase
ALT: Alanine aminotransferase
AST: Aspartate aminotransferase
BW: Body weight
Compound 3B: N^1^-(2,2’-bipyridin-6-yl)-N^8^-hydroxyoctanediamide
CREA: Creatinine
H&E: Hematoxylin and Eosin
HDAC_i_: Histone deacetylase inhibitor
IAEC: Institutional Animal Ethics Committee
LD_50_: Lethal dose 50
OECD: Organization for Economic Cooperation and Development
SD: Standard deviation
UR: Urea
